# Exploring the therapeutic use and outcome of antibody-drug conjugates in ovarian cancer treatment

**DOI:** 10.1038/s41388-025-03448-3

**Published:** 2025-05-27

**Authors:** Revu V. L. Narayana, Romi Gupta

**Affiliations:** 1 Department of Biochemistry and Molecular Genetics, The University of Alabama at Birmingham, Birmingham, AL USA; 2 O’Neal Comprehensive Cancer Center, The University of Alabama at Birmingham, Birmingham, AL USA

**Keywords:** Ovarian cancer, Targeted therapies

## Abstract

Ovarian cancer remains a leading cause of cancer-related deaths due to late-stage diagnosis and treatment resistance. While surgery and chemotherapy are standard treatments, challenges such as platinum resistance, tumor heterogeneity, and limited therapeutic options persist. Antibody-drug conjugates (ADCs) have emerged as a promising therapeutic strategy for treating ovarian cancer (OC), particularly in cases of platinum-resistant ovarian cancer (PROC). In OC, various ADCs targeting antigens such as folate receptor alpha (FRα), trophoblast cell surface antigen 2 (TROP-2), mesothelin (MSLN), sodium-dependent phosphate transport protein 2B (NaPi2b), and human epidermal growth factor receptor 2 (HER2) have shown encouraging preclinical results and significant clinical activity. However, challenges like antigen heterogeneity, off-target toxicity, and resistance mechanisms remain. This review highlights the current ADCs used in the clinic for the treatment of ovarian cancer, their challenges, and the future potential of ADC-based therapies in overcoming resistance and improving patient outcomes.

## Introduction

Ovarian cancer (OC) is an aggressive gynecological malignancy, characterized by a high degree of inter- and intra-tumoral heterogeneity at both the cellular and molecular levels. It is often diagnosed at advanced stages, leading to a high mortality rate [1, 2]. There are various types of OC, including epithelial ovarian cancer (EOC), germ-cell ovarian tumors, sex cord-stromal tumors, small-cell carcinomas, and ovarian carcinosarcomas [3]. EOC accounts for ~85–90% of all OC cases [3]. Treatment for OC typically involves cytoreductive surgery followed by combination chemotherapy with platinum agents and taxane derivates [4, 5]. The initial treatment for OC has an 80–90% success rate, but many patients experience recurrence and develop resistance to chemotherapy, resulting in a poor prognosis and a 5-year survival rate of <35% [6].

Therapeutic advancements in OC have emerged with the development of targeted therapies, such as anti-angiogenic agents and poly (ADP-ribose) polymerase (PARP) inhibitors (PARPi) [7]. Bevacizumab, an anti-angiogenic agent that blocks vascular endothelial growth factor (VEGF), was the first targeted therapy approved by the US FDA for OC. It has shown efficacy in patients with newly diagnosed, platinum-sensitive, and platinum-resistant recurrent OC [8, 9]. PARPi represents the second major class of targeted therapies and has recently gained prominence, especially for women with BRCA1 or BRCA2 mutations or those with a deficient homologous recombination (HR) repair pathway. Currently, three PARP inhibitors- Olaparib, Niraparib, and Rucaparib are FDA-approved for the management of OC [10, 11]. Despite promising clinical outcomes with PARP inhibitors, their adverse toxicity profile, along with drug resistance and tumor recurrence, remain significant challenges [12–14]. Thus, there is an urgent need for new therapeutic options and the identification of additional biomarkers to improve outcomes for OC patients.

## Antibody-drug conjugate (ADC) therapy in cancer

The development of monoclonal antibodies (mAbs) in the 1970s, following the hybridoma technique, revolutionized cancer treatment by enabling targeted therapies [15]. mAbs bind specifically to antigens on cancer cells, reducing off-target effects and enhancing therapeutic precision. This mechanism allows mAbs to either alter signaling pathways or stimulate the immune response to attack cancer cells. Over 100 mAbs have been FDA-approved for various diseases, including cancer and autoimmune disorders [16]. The evolution of mAb technology led to the creation of ADCs, which combine mAbs with cytotoxic drugs. This concept, originating from Paul Ehrlich’s “magic bullet” theory, allows for the selective delivery of toxic agents to targeted tumor cells [17]. ADCs used for therapy are a blend of chemotherapy and immunotherapy, featuring a potent cytotoxic payload linked to an antibody, which provides tumor-specific targeting and extends the antibody’s half-life in circulation. This dual-action approach improves clinical outcomes and offers a better quality of life for patients compared to traditional chemotherapy [18–20].

The first ADC was developed in the 1950s, with methotrexate linked to polyclonal rodent Ig targeting leukemia cells. Clinical trials for ADCs began in the 1980s [21], but their success was limited due to immunogenicity, low potency, and inadequate target selectivity [22]. Over time, the development of next-generation ADCs using humanized antibodies reduced immunogenicity. In 2000, under an accelerated approval process, the US FDA approved the first ADC, Mylotarg (gemtuzumab ozogamicin), which targets CD33 for the treatment of relapsed acute myeloid leukemia (AML) [23, 24]. However, its use was halted due to concerns related to adverse toxic effects, particularly myelosuppression [25]. It was further reapproved for use in 2017, albeit at a lower dose. Later, in 2011, the FDA approved Brentuximab vedotin, a CD30-targeted ADC, for the treatment of relapsed and/or refractory classical Hodgkin lymphoma (HL) and systemic anaplastic large cell lymphoma (ALCL) [26], followed by Ado-trastuzumab emtansine (T-DM1) in 2013, a HER2-targeted ADC, for the treatment of trastuzumab-resistant metastatic breast cancer [27]. Since the approval of the first ADC, several ADCs have been FDA-approved, and more than 100 ADCs are currently at various stages of clinical trials [28] (Table 1).

**Table 1:** ADCs for cancer treatment.

ADC drug	Brand name	Company	Target antigens	Tumor type
Gemtuzumab Ozogamicin	Mylotarg	Pfizer Inc.	CD33	Acute Myeloid Leukemia
Brentuximab Vedotin	Adcetris	Seagen Inc.	CD30	Lymphoma, Hodgkin Lymphoma, Diffuse Large Cell Lymphoma
Trastuzumab Emtansine	Kadcyla	Genentech Inc.	HER2	Breast Cancer
Inotuzumab Ozogamicin	Besponsa	Pfizer Inc.	CD22	Acute Lymphoblastic Leukemia
Moxetumomab Pasudotox	Lumoxiti	AstraZeneca	CD22	Hairy Cell Leukemia
Polatuzumab Vedotin	Polivy	Genentech Inc.	CD79b	Diffuse Large B-cell Lymphoma
Enfortumab Vedotin	Padcev	Astellas Pharma US Inc.	Nectin-4	Urothelial Carcinoma
Trastuzumab Deruxtecan	Enhertu	AstraZeneca and DaiichiSankyo	HER2	Breast Cancer, Gastric Cancer, Non-Small Cell Lung Cancer, Solid Tumors
Sacituzumab Govitecan	Trodelvy	Gilead Sciences Inc.	TROP2	Breast Cancer
Loncastuximab Tesirine	Zynlonta	ADC Therapeutics SA	CD19	Diffuse Large B-cell Lymphoma
Tisotumab Vedotin	Tivdak	Seagen Inc.	TF	Cervical Cancer
Mirvetuximab Soravtansine	Elahere	AbbVie Inc.	FRα	Ovarian Cancer, Fallopian Tube Cancer, Peritoneal Cancer
Datopotamab Deruxtecan (Dato-DXd)	Datroway	AstraZeneca and Daiichi Sankyo	TROP2	Breast Cancer

## Key components of ADCs and mechanisms-of-action

The key components of an ADC include a tumor-specific antibody, a stable chemical linker (either cleavable or non-cleavable), and a potent cytotoxic drug (payload) [29]. An ideal ADC remains stable in blood circulation, accurately targets the therapeutic site, and effectively releases the cytotoxic payload [29]. Each of these elements plays a crucial role in determining the overall efficacy and safety of ADCs.

### Target antigen and associated antibody selection

A key aspect of ADC development is identifying a unique antigenic target for the mAb component. The ideal antigen for ADCs should be highly expressed on the surface of target cancer cells, with low or no expression on normal cells. This ensures selective internalization of the ADC-antigen complex by cancer cells and precise release of the cytotoxic payload [30, 31]. To date, over 800 unique antibody-based molecules/cells targeting more than 300 distinct antigens have been developed or approved for therapeutic use across multiple diseases [32].

The primary characteristics of mAbs in ADC development include minimal immunogenicity, target specificity (with sufficient antigen specificity and affinity), efficient internalization, and a long half-life in circulation. In ADCs, immunoglobulin G (IgG), particularly the IgG1 subclass, is often chosen due to its abundance and proven therapeutic utility. The subclasses of IgG (IgG1, IgG2, IgG3, or IgG4) play an important role in determining the functions of the antibody Fc domain. This domain governs key aspects of ADCs, such as effector functions (e.g., ADCC, ADCP), antigen affinity, and biodistribution [33].

The size of the antibody is also critical in determining its ability to penetrate tumor tissues. Full-length IgG antibodies, with a molecular weight of ~150 kDa, often face challenges in traversing blood capillaries and the dense extracellular matrix of solid tumors [34]. While first-generation ADCs were primarily developed for hematological malignancies, current efforts focus on developing miniaturized antibodies (by removing the Fc segment) to improve the targeting of solid tumors. These smaller antibodies possess high affinity, specificity, and better penetration through blood vessels into solid tumors, thereby enhancing their therapeutic effects [35].

### Linkers

The stability of ADCs in circulation is maintained by chemical linkers, which serve as a bridge between the cytotoxic drugs and mAbs. Linker chemistry and the site of conjugation significantly impact ADC performance in terms of stability, pharmacokinetics, pharmacodynamics, and cytotoxicity. The ideal linker should prevent ADC aggregation, promote payload release at the targeted sites, and limit premature release. Linkers can be categorized as either cleavable or non-cleavable, with functional groups such as disulfides, hydrazones, and thioethers [36, 37].

Cleavable linkers rely on the physiological environment (e.g., low pH, proteolysis, or elevated intracellular glutathione levels) to release the payload from the ADC. Non-cleavable linkers form non-reducible connections with amino acid residues of the mAb, making them more stable in circulation. Non-cleavable linkers also require internalization for the payload to be released via lysosomal breakdown of the mAb [37, 38]. Linkers can also affect the payload’s electrical charge, influencing the payload’s ability to diffuse across membranes and thus imparting or preventing a “bystander effect” [39].

### Cytotoxic payloads

Cytotoxic payloads, or “warheads,” are the ultimate effectors of ADCs. These small-molecule drugs, typically weighing between 300 and 1000 Da and possess sufficient potency to eradicate tumors at low concentrations, even at nano- or picomolar IC50 values [40, 41]. Currently, the most common cytotoxic payloads for ADCs include DNA-damaging compounds, strong tubulin inhibitors, and immunomodulators (Table 2).

**Table 2:** Class of payloads associated with ADCs and their mechanism-of-action.

Class of payload	Name	Mechanism of action
Tubulin inhibitors	Auristatins Ex: MMAE (vedotin) MMAF (mafodotin)	Inhibits tubulin polymerization by targeting the β-subunits of tubulin dimer, thereby disrupting microtubule growth.
	Maytansinoids Ex: DM1: emtansine/mertansine DM4: soravtansine/ravtansine	Blocks the polymerization of tubulin dimer and inhibits the formation of mature microtubules.
	Tubulysins Ex: AZ13599185	Inhibits tubulin polymerization.
DNA damaging agents	Calicheamicins	Promotes DNA double-strand break by binding to the minor groove of DNA and inducing strand cleavage.
	Duocarmycins	Promotes DNA alkylation by binding to the minor groove of DNA and alkylating the nucleobase adenine at the N3 position, leading to modification of DNA structure.
	Exatecans	Inhibits topoisomerase I enzyme by binding to the topoisomerase I-DNA complex and preventing DNA re-ligation, leading to DNA damage.
	Pyrrolobenzodiazepines	Promotes DNA alkylation and interstrand DNA cross-links, leading to disrupting DNA structure.
Immunomodulators	TLR agonizts	Stimulates innate immunity, leading to production of cytokines and proinflammatory molecules to effectively kill the cancer cells.
	STING agonizts	Promotes activation of type I interferons and other inflammatory cytokines, leading to anti-tumor activity.

The Drug-to-Antibody Ratio (DAR) represents the average number of payload molecules conjugated to each antibody in an ADC. A lower DAR may indicate lower potency, while a higher DAR is often associated with increased therapeutic activity. However, a high DAR can sometimes compromise ADC efficacy due to altered biodistribution (e.g., accumulation in non-target organs), accelerated drug clearance, and impaired antigen binding, depending on the payload properties [42, 43].

**Fig. 1:**
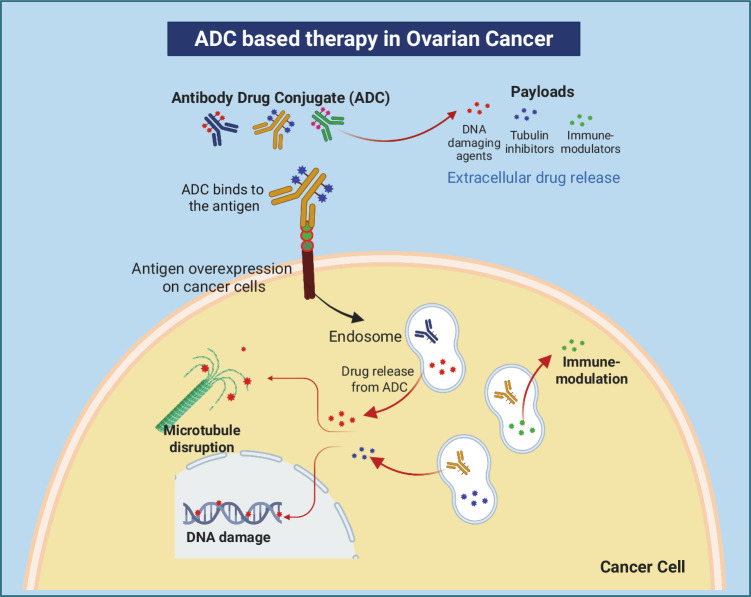
A model illustrating the different mechanisms by which various antibody-drug conjugates (ADCs) function as therapeutic agents in ovarian cancer. Antibody-drug conjugates (ADCs) consist of an antibody bound to a cytotoxic drug through a linker. The antibody targets specific cancer cell antigens, binding to them and entering the cell via receptor-mediated endocytosis. Once inside, the cytotoxic drug is released, typically through enzymatic cleavage, and it kills the cancer cell by disrupting essential processes like DNA replication, microtubule disruption, and immune modulation, among other mechanisms. This targeted approach allows for the delivery of potent chemotherapy specifically to tumor cells with minimum effect on normal tissues.

Since ADCs use monoclonal antibodies to precisely deliver potent chemotherapy to tumor cells, they are shown to have minimal off-target effects and enhanced tumor suppression [44]. They are also administered intravenously to prevent the degradation of the mAb by gastric acid and other proteolytic processes [45]. Mechanistically, once ADCs bind to the antigen on target cells, they are internalized primarily via receptor-mediated endocytosis, which occurs primarily through clathrin-mediated or caveolae-mediated endocytosis. In rare instances, internalization may occur via pinocytosis [46–48]. The internalized ADCs form early endosomes, which mature into late endosomes and fuse with lysosomes, creating an acidic environment. This acidic environment, combined with lysosomes rich in proteases such as cathepsin-B and plasmin, facilitates the cleavage of the ADCs, leading to the optimal release of the cytotoxic drug into the cytoplasm. Once released, the drug disrupts cellular processes, triggers apoptosis, and ultimately causes cell death [49, 50] (Fig. 1).

In addition to the anticancer activity, ADCs also trigger immune-mediated cell responses, such as antibody-dependent cellular cytotoxicity (ADCC), antibody-dependent cellular phagocytosis (ADCP), and complement-dependent cytotoxicity (CDC), which ultimately contribute to tumor clearance [51–53] (Fig. 1).

## ADCs for ovarian cancer treatment

**Fig. 2:**
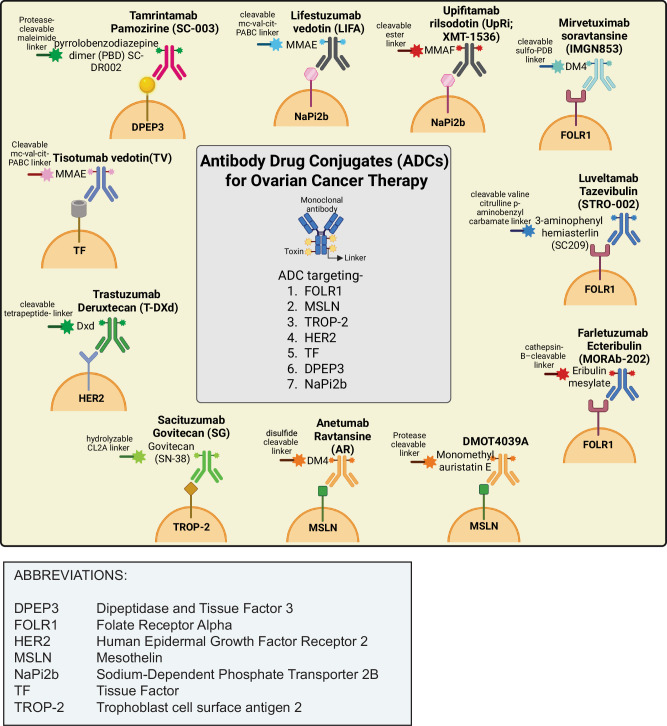
Image showing different types of ADCs used in ovarian cancer therapy. Several types of antibody-drug conjugates (ADCs) are being explored for ovarian cancer therapy in clinic. These ADCs target different cell surface antigens, such as folate receptor-alpha (FRα), HER2, TROP-2, and MSLN, among others, and carry different types of cytotoxic payloads to induce cell death upon release in tumor cells.

Numerous clinical trials have provided evidence demonstrating the manageable toxicity profile and promising clinical efficacy of ADCs in patients with ovarian cancer. Several ADCs have been developed to target various antigens in OC treatment, including folate receptor alpha (FRα), trophoblast cell surface antigen 2 (TROP-2), mesothelin (MSLN) sodium-dependent phosphate transport protein 2B (NaPi2b), human epidermal growth factor receptor 2 (HER2), dipeptidase 3 (DPEP3), and tissue factor (TF), utilizing a wide range of cytotoxic payloads [54]. Below, we describe ADCs that are currently being evaluated in clinical trials for treating ovarian cancer patients (Fig. 2 and Table 3).

**Table 3:** Clinical trials with ADCs in Ovarian Cancer.

Target	ADC	Clinical trial number and reference number	Phase and study design	Dose and regimen for ADC	Objective	Survival outcomes and responses	Adverse effects
FRα	Mirvetuximab Soravtansine (MIRV) (IMGN853)	NCT01609556 PMID: 28440955 [61] PMID: 28029313 [62]	Phase I dose-escalation and expansion study with open-label cohort	6 mg/kg; every 3 weeks, administered intravenously	Safety and clinical activity, ORR, PFS, and DOR	For expansion phase- -median PFS:4.8 -ORR: 26% -median DOR: 19.1 weeks	Diarrhea, blurred vision, nausea, and fatigue
		NCT02631876 (FORWARD I trial) PMID: 29424243 [63] PMID: 33667670 [64]	Phase III, randomized trial. MIRV compared with chemotherapy (paclitaxel, pegylated liposomal doxorubicin, or topotecan).	6 mg/kg; Every 3 weeks administered intravenously	PFS, ORR, OS, and DOR	Superior outcomes for MIRV over chemotherapy were observed in all secondary endpoints in the FRα high population, including improved ORR (24% vs. 10%)	Nausea, diarrhea, and blurred vision, less myelosuppression, less peripheral neuropathy, and no alopecia.
		NCT04296890 (SORAYA trial) PMID: 36716407 [65] PMID: 38858103 [66] PMID: 37212825 [67]	Phase II, Single-arm trial	6 mg/kg; every 3 weeks, administered intravenously	ORR, DOR, and TRAE	-median DOR: 6.9 months, -median PFS: 4.3 months -median OS: 15 months -ORR: 32.4%	Blurred vision, keratopathy, and nausea
		NCT04209855 (MIRASOL trial) PMID: 38055253 [68]	Open-labeled randomized phase III clinical trial comparing MIRV with chemotherapy	6 mg/kg; every 3 weeks administered intravenously	PFS, OS, and objective response	-median OS of 16.46 months with MIRV vs. 12.75 months with chemotherapy, -median PFS of 5.62 months with MIRV vs. 3.98 months with chemotherapy, - ORR of 42.3% with MIRV vs. 15.9% with chemotherapy.	Blurred vision, keratopathy, abdominal pain, and fatigue in MIRV MIRV-treated group.
		NCT02606305 (FORWARD II trial) PMID: 36736157 [69]	Multi-arm, phase Ib/II trial	MIRV-6mg/kg; every 3 weeks, and bevacizumab (15 mg/kg) once every 3 weeks, administered intravenously	Safety and clinical activity of MIRV in combination with bevacizumab	-median PFS was 8.2 months, -ORR was 44%, including 5 CRs -median DOR: 9.7 months	Diarrhea, blurred vision, and nausea
	Luveltamab Tazevibulin (STRO-002-GM1)	NCT03748186 PMID: 36459691 [71]	Phase I dose expansion cohort	4.3 mg/kg or 5.2 mg/kg once every 3 weeks, administered intravenously	Safety and efficacy	-Median DOR was 13 months in the 4.3 mg/kg group and 5.4 months in the 5.2 mg/kg group -median PFS was 6.1 months in the 4.3 mg/kg group and 6.6 months in the 5.2 mg/kg group, -ORR was 31.3% in the 4.3 mg/kg group and 43.8% in the 5.2 mg/kg group	Neutropenia, arthralgia, anemia
	Farletuzumab Ecteribulin (MORAb-202)	NCT03386942 PMID: 33926914 [74]	Dose escalation phase I trial	0.3 to 1.2 mg/kg once every 3 weeks, administered intravenously	dose-limiting toxicities, safety, tumor responses, pharmacokinetics, and pharmacodynamics	Total: 22 patients -CR was seen in 1 -PR was seen in 9 -SD was seen in 8	Leukopenia and neutropenia
TROP2	Sacituzumab Govitecan (SG)	NCT01631552 PMID: 33741442 [79]	Phase I/II IMMU-132-01 basket trial	(8, 10, 12, or 18 mg/kg) On days 1 and 8 of 21-day cycles administered intravenously	ORR, DOR, clinical benefit rate, PFS, and OS	-Overall safety population was 1.6% for EOC	Nausea, diarrhea, fatigue, alopecia, and neutropenia
Mesothelin (MSLN)	Anetumab Ravtansine	NCT02751918 PMID: 36564099 [83]	Phase Ib clinical trial	6.5 mg/kg every three weeks administered intravenously	Safety, pharmacokinetics, and efficacy	-ORR: 27.7% -CR: 1 -PR: 17 -median DOR: 7.6 months -median PFS: 5.0 months	Nausea, decreased appetite, fatigue, diarrhea, and corneal disorder
		NCT03587311 [85]	Phase II randomized trial	2.2 mg/kg weekly on 28-day cycle administered intravenously	Safety and efficacy of the combination of AR/bevacizumab (ARB) vs. weekly paclitaxel/bevacizumab in PROC	-PFS of 5.3 months for the combination of AR and bevacizumab, compared to 9.6 months for bevacizumab and paclitaxel - ORR of 18% for the combination of AR and bevacizumab, compared to 55% for bevacizumab and paclitaxel	Thrombocytopenia, fatigue, increased ALT and AST, peripheral neuropathy
	DMOT4039A	NCT01469793 PMID: 26823490 [84]	Phase I clinical trials	2.4 mg/kg every three weeks administered intravenously	Safety and pharmacokinetics	Total OC patients: 31 -PR observed in 4 out of 31	Hyperglycemia, hypophosphatemia, gastrointestinal, and constitutional
Human Epidermal growth factor 2 (HER2)	Trastuzumab Deruxtecan (T-DXd)	NCT04482309 (DESTINY-PanTumor02 trial) PMID: 37870536 [92]	Open-label phase II study	5.4 mg/kg every three weeks administered intravenously	ORR, safety, DOR, PFS, and OS	In all patients, -ORR: 37.1% -median DOR: 11.3 months -median PFS: 6.9 months -median OS: 13.4 months	Grade ≥3 TRAE, drug-related interstitial lung disease, with three deaths
Sodium-dependent phosphate transporter 2B (NaPi2b)	Lifestuzumab Vedotin (LIFA)	NCT01911598 PMID: 31540980 [95]	Phase I clinical study	0.2 to 2.8 mg/kg dose on a tri-weekly schedule administered intravenously	Safety, tolerability, and preliminary antitumor activity	At an active dose ≥ 1.8 mg/kg, PR was 11/24 (46%)	Fatigue, nausea, decreased appetite, vomiting, and peripheral sensory neuropathy. Most common ≥3 TRAE included neutropenia, anaemia, and pneumonia.
Sodium-dependent phosphate transporter 2B (NaPi2b)	Lifestuzumab Vedotin (LIFA)	NCT01991210 PMID: 29401246 [98]	Open-label clinical trial	2.4 mg/kg on a tri-weekly schedule LIFA was administered intravenously or pegylated liposomal doxorubicin (PLD)	Comparing ADC with respect to standard of care	PFS was of 5.3 in LIFA vs. 3.1 months in PLD, and the ORR was 34% in LIFA vs. 15% in PLD	Severe toxicities and ≥3 TRAE
Sodium-dependent phosphate transporter 2B (NaPi2b)	UpRi; XMT-1536	NCT03319628 [101]	Phase Ib/2 clinical trial	36–43 mg/m^2^ on every 4 weeks injected intravenously	Anti-tumor activity	ORR of 32% with CR in 2 PROC patients	High ALT, nausea, fatigue, and pyrexia
Dipeptidase 3 (DPEP3)	Tamrintamab Pamozirine (SC-003)	NCT02539719 PMID: 32513564 [103]	Phase 1a/1b clinical study	0.025 to 0.4 mg/kg every 3 weeks, administered intravenously	Safety, tolerability, pharmacokinetics, and preliminary antitumor activity	-ORR: 4% -No durable response	Fatigue, nausea, decreased appetite, pleural effusion, abdominal pain, and peripheral edema
Tissue Factor	Tisotumab Vedotin (TV)	NCT02001623 (innovaTV 201 trial) PMID: 30745090 [106]	Phase 1–2, open-label, dose-escalation and dose-expansion study	0·3 and 2·2 mg/kg once every 3 weeks, administered intravenously	Safety, tolerability, pharmacokinetic profile, and antitumour activity	-ORR: 15·6% across various tumor types	Grade 3 TRAE, including type 2 diabetes mellitus, mucositis, and neutropenic fever, including nine deaths.

## Folate receptor alpha (FRα)

FRα is a GPI-anchored glycoprotein encoded by the FOLR1 gene, and it is widely expressed in various non-malignant tissues. Its primary function is to transport folates into the cells, which are essential for cellular proliferation and DNA synthesis. Upregulation of FRα is associated with an increased demand for enzymatic reactions involved in one-carbon metabolism, a critical hallmark of carcinogenesis [55]. FRα is expressed in many OC cases, with expression reported in 76% of high-grade serous OC (HGSOC), according to the Ovarian Tumor Tissue Analysis (OTTA) consortium [56]. Additionally, a previous study reported that FRα expression remains unchanged after chemotherapy, making it an attractive candidate for targeted therapy [57].

## ADCs targeting folate receptor alpha (FRα) in ovarian cancer

### Mirvetuximab soravtansine

Mirvetuximab Soravtansine (MIRV) or IMGN853 is an anti-FRα ADC and was developed by ImmunoGen Inc. It consists of humanized IgG1 antibody conjugated to a DM4 payload via a charged, cleavable sulfo-PDB linker with a 3.5:1 DAR. DM4 is a maytansinoid derivative and functions as an antitubulin agent. After cellular internalization, it arrests the G2-M phase of the cell cycle, leading to cell death. Additionally, the S-methyl-DM4 payload used is electrically neutral and lipophilic, enabling it to diffuse across biomembranes and exert a bystander effect [58]. An early preclinical study of IMGN853 demonstrated potent cytotoxic activity against FRα-expressing tumors in OC xenografts. The bystander effect was also observed in FRα-negative tumors [59]. Another study showed that when IMGN853 was combined with established OC treatments, including carboplatin, doxorubicin, and bevacizumab, there was rapid disruption of tumor microvasculature [60].

Mirvetuximab Soravtansine (MIRV) monotherapy was tested for clinical safety in different ovarian cancer clinical trials with FRα expression and recurrent disease as described below. The phase 1 trial (NCT01609556) enrolled 44 patients with FRα-positive epithelial ovarian cancer (EOC) and tested MIRV doses ranging from 0.15 to 7.0 mg/kg every three weeks or weekly. Two patients showed partial responses (PR). The most common treatment-related adverse events (TRAEs) were fatigue, blurred vision, and diarrhea, mostly mild (grade 1 or 2). Dose-limiting toxicities occurred at higher doses, including grade 3 hypophosphatemia at 5.0 mg/kg and punctate keratitis at 7.0 mg/kg. A recommended phase 2 dose of 6.0 mg/kg every three weeks was established [61, 62]. During the expansion phase of the trial, 46 FRα-positive platinum resistant ovarian cancer (PROC) patients who had undergone up to five prior lines of therapy, when treated with IMGN853 at 6.0 mg/kg once every three weeks achieved an overall response rate (ORR) of 26%, median duration of response (DOR) of 19.1 weeks, and median progression-free survival (PFS) of 4.8 months. It was also observed that the patients with fewer than three lines of prior treatments had a higher ORR of 39%, PFS of 6.7 months, and median DOR of 19.6 weeks. The most common observed TRAEs in this study were diarrhea, blurred vision, nausea, and fatigue [61, 62].

The FORWARD I trial (NCT02631876), a phase 3 randomized study, compared MIRV (given to 243 patients at dose of 6 mg/kg every 3 weeks) to chemotherapy (given to 109 patients) in PROC patients who were FRα-positive and had 1 to 3 prior treatments. No significant difference in PFS was found between MIRV and chemotherapy (HR 0.98; *P* = 0.897) treated group, with median PFS of 4.1 (MIRV) vs. 4.4 (chemotherapy) months. However, MIRV showed superior outcomes in high-FRα expressing patients, with higher ORR of 24% (MIRV) vs. 10% (chemotherapy), median PFS of 4.8 (MIRV) vs. 3.3 (chemotherapy) months, and better CA-125 responses (53% in MIRV vs. 25% in chemotherapy). MIRV also had a more favorable safety profile, with fewer grade 3+ adverse events, dose reductions, and discontinuations [63, 64].

The SORAYA trial (NCT04296890), a phase 2 study, tested MIRV (administered at a dose of 6 mg/kg every 3 weeks) in 106 PROC patients with high FRα expression (≥75% of cells) who had received 1 to 3 prior therapies. Prior bevacizumab use was required. The ORR observed was 32.4%, with 5 complete responses (CR) and 29 partial responses (PR). Median DOR was found to be 6.9 months, and the final median overall survival (OS) was 15 months [65–67]. Most common TRAEs included blurred vision, keratopathy, and nausea, which led to dose delays in 33% of cases, reductions in 20% of cases, and discontinuations in 9% of cases. These results led to FDA approval of MIRV in November 2022 as the first ADC for treating FRα-positive, platinum-resistant ovarian cancer patients [65–67].

Next, in the MIRASOL trial (NCT04209855), a phase 3 study, safety and efficacy of MIRV treatment (in 227 patients administered at dose of 6 mg/kg adjusted for ideal body weight) was compared to chemotherapy (in 226 patients administered with paclitaxel, pegylated liposomal doxorubicin [PLD], or topotecan) in PROC patients who had received prior 1 to 3 prior treatments (total 453 patients) and had high FRα tumor expression. MIRV administered at a 6 mg/kg dose in 227 patients demonstrated improved outcomes, with a median OS of 16.46 months vs. 12.75 months in chemotherapy treated group, a median PFS of 5.62 vs. 3.98 months in chemotherapy treated group and a higher ORR of 42.3% vs. 15.9% as compared to chemotherapy treated group. Additionally, MIRV had favorable safety profile and fewer adverse side effects [68].

The efficacy of MIRV has also been explored in combination with immunotherapy and chemotherapy in FRα-expressing 94 PROC patients. The FORWARD II trial (NCT02606305), a multi-arm, phase Ib/II study, evaluated the safety and clinical activity of MIRV in combination with bevacizumab. The trial showed an ORR of 44%, with 5 CR, a median DOR of 9.7 months, and a median PFS of 8.2 months. Most common TRAEs included blurred vision, diarrhea, and nausea. These findings suggested that the combination of MIRV and bevacizumab can effectively be used for treating FRα-expressing PROC patients in the clinic [69]. Future trials, such as phase III GLORISA trial, are evaluating the effect of MIRV in combination with bevacizumab vs. bevacizumab alone as maintenance therapy in patients with FRα-high PSOC patients [70]. These results indicate the importance of conducting future trials with MIRV in combination other agents to determine its effectiveness in treating both platinum-resistant ovarian cancer (PROC) and platinum-sensitive ovarian cancer (PSOC) patients in clinic.

### Luveltamab tazevibulin (STRO-002 or Luvelta)

STRO-002, also called Luvelta, was developed by Sutro Biopharma. It was designed to have potent and specific cytotoxicity against FRα-expressing tumor cells. STRO-002 consists of a high-affinity anti-FRα antibody conjugated to 3-aminophenyl hemiasterlin warhead (SC239) via cleavable linker with a DAR of 4. This warhead targets tubulin. Both in vitro and in vivo preclinical studies on OC cell lines demonstrated that STRO-002 has promising targeted delivery, enhanced therapeutic potential with superior stability, and favorable pharmacokinetic properties. Additionally, STRO-002 induced significant tumor growth inhibition in IGROV and OVCAR3, OC cell line xenograft models, as well as in PDX models. Combination treatment with carboplatin or avastin further enhanced its efficacy [71].

In a phase I dose-expansion clinical trial (STRO-002-GM1; NCT03748186), the safety and efficacy of STRO-002 were evaluated in recurrent EOC patients who had either platinum-resistant disease after 1–3 prior lines of treatment or platinum-sensitive disease after 2–3 prior lines of platinum chemotherapy. Forty-four patients were randomized in a 1:1 ratio to receive STRO-002 either at doses of 4.3 mg/kg (23 patients) or 5.2 mg/kg (21 patients), and FRα expression was assessed. The results showed ORR of 31.3% in the 4.3 mg/kg treated group and 43.8% in the 5.2 mg/kg treated group, median DOR of 13 months in the 4.3 mg/kg treated group and 5.4 months in the 5.2 mg/kg treated group and PFS of 6.1 months in the 4.3 mg/kg treated group and 6.6 months in the 5.2 mg/kg group. Most common TRAEs included neutropenia, sepsis, arthralgia, and anemia [71]. In summary, the study confirmed the clinical activity of Luvelta, and future phase II/III REFRaME trial is planned to evaluate the efficacy of Luvelta in PROC with higher FRα expression [72].

### Farletuzumab ecteribulin (MORAb-202)

MORAb-202 was developed by Eisai and Bristol Myers Squibb. It comprises of farletuzumab, an antibody targeting FRα conjugated with eribulin mesylate, a microtubule-targeting agent as the payload via a cleavable linker with a DAR of 4. The cathepsin-B–cleavable linker allows targeted delivery and activation of the cytotoxic drug within FRα-expressing tumor cells [73]. Preclinical studies using FRα-positive human cancer cell line xenograft models and PDX models demonstrated that MORAb-202 promotes significant tumor growth inhibition [73].

A dose-escalation phase I trial (NCT03386942) investigated the safety and tolerability of MORAb-202 in patients with FRα-positive solid tumors. In this trial, patients received MORAb doses ranging from 0.3 to 1.2 mg/kg every three weeks. Among 22 patients evaluated, 8 patients had stable disease, 9 patients experienced PR, and 1 patient achieved CR. Most common TRAE included leukopenia and neutropenia [74]. These findings suggested MORAb-202 antitumor activity in patients with FRα-positive solid tumors, including OC, and its use as a new treatment option for these patients. Further, in-depth studies are needed to determine its optimal clinical usage.

## Trophoblast cell surface antigen 2 (TROP-2)

Trophoblast Cell Surface Antigen 2 (TROP-2) is a transmembrane glycoprotein of the GA733 gene family, encoded by the TACSTD2 gene. TROP-2 is also known as epithelial glycoprotein 1 (EGP-1), gastrointestinal antigen 733-1 (GA733-1), membrane component surface marker 1 (M1S1), and tumor-associated calcium signal transducer 2 (TACSTD2). It plays a crucial role in calcium signal transport, which is essential for cell cycle-related signaling pathways. TROP-2 is highly expressed in most human cancers but minimally expressed in normal tissues, making it a promising target for cancer therapies [75]. Studies in ovarian cancer have shown that TROP-2 knockdown results in the inhibition of tumor growth, invasion, and metastasis [76].

## ADCs targeting trophoblast cell surface antigen 2 (TROP-2) in ovarian cancer

### Sacituzumab govitecan

Sacituzumab Govitecan (SG) is a TROP-2 targeting ADC and was developed by Immunomedics. SG consists of sacituzumab, a humanized IgG1 monoclonal antibody targeting TROP-2, conjugated with payload govitecan (SN-38; a topoisomerase inhibitor) via a cleavable CL2A linker with a 7.6 to 8.1 DAR ratio. Preclinical studies showed that SG can effectively inhibit the growth of chemotherapy-resistant OC both in in vitro and in vivo models and exert a bystander effect on TROP-2-negative OC cells [77, 78]. In a Phase I/II IMMU-132-01 basket trial (NCT01631552), SG was evaluated for safety and efficacy in various solid tumors, including epithelial ovarian cancer. It was observed that in a small group of EOC patients (*n* = 8), the overall safety population (OSP) was very low (1.6%). As a result of adverse effects, this treatment was discontinued. Thus, this trial demonstrated that SG presents significant toxicity at the dose used, and future studies are needed to optimize the dosage to determine its potential effectiveness in treating ovarian cancer (OC) [79].

## Mesothelin (MSLN)

Mesothelin (MSLN) is a GPI-anchored membrane glycoprotein expressed on mesothelial cells in the pleura, peritoneum, and pericardium and is involved in tumor differentiation [80]. It is found to be highly expressed in many solid tumors, including in 70% of OC cases, making it a potentially useful tumor-associated marker for targeted therapies [80, 81].

## ADCs targeting mesothelin (MSLN) in ovarian cancer

### Anetumab ravtansine

Anetumab Ravtansine (AR), also known as BAY 94-9343, was developed by Bayer HealthCare Pharmaceuticals in collaboration with ImmunoGen Inc. AR consists of a humanized anti-mesothelin monoclonal antibody (IgG1) conjugated to the maytansinoid DM4 via a disulfide-cleavable linker, with an average DAR of 3.2. Preclinical studies in OC models showed that AR exhibited potent and selective tumor growth inhibition in both subcutaneous and orthotopic OVCAR-3 xenograft models. Additionally, AR demonstrated a bystander effect on surrounding mesothelin-negative tumor cells [82].

In a phase Ib clinical trial (NCT02751918), a cohort of 65 mesothelin-expressing PROC patients was treated with AR in combination with pegylated liposomal doxorubicin (PLD). The maximum tolerated dose (MTD) was determined to be 6.5 mg/kg every three weeks. In the treated group, the ORR was 27.7%, including one CR, and 17 PR, median DOR was 7.6 months, and median PFS was 5.0 months [83]. The most common TRAEs included nausea, fatigue, decreased appetite, diarrhea, and corneal disorder [83]. Another Phase I clinical trial (NCT01469793) evaluated the efficacy of DMOT4039A, an antibody-drug conjugate (ADC) composed of a humanized anti-mesothelin monoclonal antibody conjugated to a monomethyl auristatin E (MMAE) warhead, a tubulin polymerization inhibitor. Among 31 patients with platinum-resistant ovarian cancer (PROC), 4 patients achieved a partial response (PR) [84]. The most common TRAEs included gastrointestinal and constitutional, highlighting the tolerability and antitumor efficacy of this ADC [84].

A more recent phase II randomized trial (NCT03587311) is evaluating the safety and activity of the combination of bevacizumab with either weekly AR treatment versus weekly paclitaxel with bevacizumab in treating PROC patients. Early results indicated that the combination of AR and bevacizumab resulted in a median progression-free survival (PFS) of 5.3 months, compared to 9.6 months for the combination of bevacizumab and paclitaxel. The objective response rate (ORR) was 18% for AR and bevacizumab, compared to 55% for bevacizumab and paclitaxel [85]. The most common treatment-related adverse events (TRAEs) for the AR and bevacizumab combination included elevated ALT/AST levels, thrombocytopenia, fatigue, and neuropathy, whereas for the bevacizumab and paclitaxel combination, the most common TRAEs were anemia, neutropenia, epistaxis, and fatigue [85]. These findings suggested that the combination of bevacizumab and chemotherapy offers a superior clinical benefit in PROC patients as compared to bevacizumab and AR, highlighting the importance of checking the efficacy of combination treatments.

## Human epidermal growth factor 2 (HER2)

Human epidermal growth factor receptor 2 (HER2) is a cell surface protein that plays a key role in regulating cell growth, division, and survival. It has been observed that HER2, when overexpressed or amplified, can drive uncontrolled cell proliferation, contributing to the development and progression of various cancers, including breast, gastric, and ovarian cancers [86]. Moreover, in ovarian cancer, HER2 overexpression has been linked to a more aggressive disease and poorer prognosis, making it a potential target for effective ovarian cancer therapy [87, 88].

## ADCs targeting human epidermal growth factor 2 (HER2) in ovarian cancer

### Trastuzumab deruxtecan (T-DXd)

T-DXd is a HER2-targeting ADC and was developed by Daiichi Sankyo and AstraZeneca. It consists of a humanized IgG1 anti-HER2 monoclonal antibody conjugated with a potent topoisomerase I inhibitor DXd via tetrapeptide-based cleavable linker, which enables the targeted delivery of the cytotoxic agent to tumor cells expressing HER2. In pre-clinical studies, T-DXd showed significantly greater in vitro efficacy on HER2-overexpressing primary and metastatic ovarian tumors and demonstrated bystander killing of HER2-negative tumor cells. In in vivo assays, T-DXd also significantly inhibited tumor growth and extended survival in HER2-overexpressing xenograft models [89]. T-DXd has also been approved by US FDA for the treatment of HER2-positive breast cancer and HER2-positive gastric cancer [90, 91].

An open-label phase II trial (NCT04482309)- DESTINY-PanTumor02 was conducted to evaluate the efficacy and safety of T-DXd in 267 patients, including 40 ovarian cancer patients with select HER2-expressing solid tumors that were locally advanced, metastatic, or inoperable. T-DXd was administered at a dosage of 5.4 mg/kg every three weeks in patients with ≥1 systemic treatment or without treatment. In all patients, total ORR was 37.1%, median DOR was 11.3 months, median PFS was 6.9 months, and OS was 13.4 months. In HER2 overexpressing patients, ORR was 61.3%, median DOR was 22.1 months, median PFS was 11.9 months, and OS was 21.1 months. Additionally, ORR for ovarian cancer was observed to be 45%, and the ones with higher HER2 expression were 63%. Half of the patients experienced TRAEs that were grade 3 or higher. In this multi-center phase II trial, T-DXd showed significant clinical activity, offering sustained benefits to patients overexpressing HER2 [92].

## Sodium-dependent Phosphate Transporter 2B (NaPi2b)

NaPi2b, is one of three members of the SLC34 family of type II sodium-dependent phosphate transporters and is encoded by the SLC34A2 gene. It plays a vital role in whole-body phosphate homeostasis by facilitating phosphate transport across epithelial cells [93]. NaPi2b is also observed to be expressed in various solid tumors, including OC. Therefore, it is considered as a promising biomarker for treatment and patient selection in ovarian cancer [94].

## ADCs targeting sodium-dependent phosphate transporter 2B (NaPi2b) in ovarian cancer

### Lifestuzumab vedotin (LIFA)

Lifastuzumab vedotin (LIFA), also referred to as DNIB0600A and RG-7599, was developed by Genentech/Roche. LIFA consists of a humanized anti-NaPi2b antibody conjugated to the monomethyl auristatin E (MMAE) warhead, which is a microtubule inhibitor, via a cleavable maleimidocaproyl-valyl-citrullinyl-p-aminobenzyloxycarbonyl (mc-val-cit-PABC) linker, with an average DAR of 3–4 [95]. In early preclinical studies, LIFA efficacy was evaluated using mouse OC xenograft models, while its toxicity was assessed in rats and cynomolgus monkeys. This study demonstrated that appropriate dosing of LIFA could achieve significant efficacy across a variety of tumor xenografts [96].

In a phase I clinical study (NCT01911598), the safety, tolerability, and preliminary antitumor activity of LIFA were evaluated in patients with NSCLC and PROC. LIFA was administered intravenously, starting at a dosage of 0.2 to 2.4 mg/kg on a tri-weekly schedule. At a dose of ≥1.8 mg/kg, PR was observed in 11 of 24 (∼46%) patients with PROC, particularly in patients with high NaPi2b expression. The most common adverse events included fatigue, nausea, decreased appetite, vomiting, and peripheral sensory neuropathy [95].

Further, phase 1b study was conducted to assess safety, tolerability, and pharmacokinetics of LIFA with a combination of carboplatin (with or without bevacizumab) in recurrent PSOC. LIFA was administered at the recommended phase 2 dose (RP2D) of 2.4 mg/kg in combination with carboplatin AUC6 (cycles 1–6), with or without bevacizumab used at the dose of 15 mg/kg. The median PFS observed for combination therapy was 10.71 months, with confirmed complete/partial responses seen in 59% of the patients [97]. All patients experienced ≥1 TRAE, most common being neutropenia, peripheral neuropathy, thrombocytopenia, nausea, fatigue, anemia, diarrhea, vomiting, hypomagnesaemia, increased aspartate aminotransferase and alanine aminotransferase, and alopecia. Of note, 83% patients experienced grade ≥ 3 TRAEs, and 22% patients experienced serious TRAEs [97]. This study concluded that LIFA can be safely used in combination with carboplatin with or without bevacizumab for PSOC patients.

Another randomized, phase II open-label clinical trial (NCT01991210) was conducted to compare the efficacy of LIFA with pegylated liposomal doxorubicin (PLD) in 95 PROC patients. Forty-seven patients received LIFA (2.4 mg/kg, every three weeks) and 48 patients received PLD (40 mg/kg, every four weeks). The medium PFS was 5.3 months for LIFA and 3.1 months for PLD, and the ORR was 34% for LIFA and 15% for PLD-treated group. This ADC was discontinued as it displayed short response duration [98]. This trial concluded that both response rate and response durability are important, underscoring the need for better evaluation of the long-term efficacy of LIFA in the treatment of OC.

### Upifitamab rilsodotin (UpRi; XMT-1536)

XMT-1536, also called UpRi, is a first-in-class Dolaflexin ADC (a novel platform featuring high drug loading and a controlled bystander effect) targeting NaPi2b and was developed by Mersana Therapeutics. XMT-1536 is comprised of a humanized IgG1 monoclonal anti-NaPi2b antibody conjugated with the microtubule inhibitor monomethyl auristatin F (MMAF) via a cleavable ester linker with an average DAR of 10 [99, 100]. MMAF is a potent tubulin polymerization inhibitor. In phase 1, clinical trial (NCT03319628), XMT-1536 was evaluated in 31 NaPi2b-positive OC patients, who showed an ORR of 32% with CR in 2 PROC patients. Based on these results, XMT-1536 is now being tested in an ongoing phase 2 UPLIFT (NCT03319628) trial with PROC patients. The outcome of this trial will help develop new ADCs for treating NaPi2b-positive ovarian cancer patients [101].

## Dipeptidase 3 (DPEP3)

DPEP3 is a member of the GPI-anchored dipeptidase family of proteins, primarily involved in hydrolyzing dipeptides into free amino acids [102]. Preclinical studies have shown that most normal tissues express DPEP3 at low or negligible levels, while elevated expression is seen in epithelial OC cells and tumor-initiating cells in OC PDX models, making it a promising target for OC therapy [103].

## ADCs targeting dipeptidase 3 (DPEP3) in ovarian cancer

### Tamrintamab pamozirine (SC-003)

Tamrintamab Pamozirine, also referred to as SC-003, was initially developed by AbbVie Stemcentrx LLC. SC-003 consists of a humanized IgG1 monoclonal antibody, SC-Mab003, conjugated with SC-DR002, a cytotoxic pyrrolobenzodiazepine (PBD) dimer, via a plasma-stable protease-cleavable maleimide linker. A phase 1a/1b clinical study (NCT02539719) was conducted to evaluate the safety/tolerability, pharmacokinetics, and antitumor effect of SC-003 alone or in combination with budigalimab (an antibody targeting PD-1), in platinum-resistant/refractory EOC patients. Seventy-four DPEP3-positive patients were recruited for the study. Among them, 29 patients received SC-003 at doses ranging from 0.025 to 0.4 mg/kg as part of the dose-escalation cohort. An additional 45 patients were included in the dose-expansion cohort. Furthermore, 3 patients were treated with SC-003 in combination with budigalimab (500 mg/kg, administered every four weeks). This study discovered that ORR in SC-003-treated patients was low, only about 4%, and lacked durability, indicating low antitumor activity. The most frequently observed TRAEs included fatigue, nausea, decreased appetite, pleural effusion, abdominal pain, and peripheral edema [103]. These studies highlight the importance of in-depth testing of the novel ADC to identify the most suitable ones to be used for OC patient treatment.

## Tissue factor (TF)

Tissue Factor (TF), also known as coagulation factor III (F3), thromboplastin, or CD142, is a transmembrane glycoprotein that is widely known to play a role in initiating extrinsic blood coagulation. It is overexpressed in a variety of solid tumors [104]. Notably, a significant proportion of ovarian cancer patients overexpress tissue factor [105], making it promising target for the treatment of ovarian cancer.

## ADCs targeting tissue factor (TF) in ovarian cancer

### Tisotumab vedotin (TV)

Tisotumab vedotin (TV) is a currently FDA-approved ADC for treating metastatic cervical cancer and is co-developed by Genmab and Pfizer. It consists of a human IgG1-kappa mAb-tisotumab targeting tissue factor, linked to the microtubule-disrupting agent MMAE (a potent tubulin polymerization inhibitor) through a protease-cleavable valine-citrulline linker with DAR of 4.1 [100]. InnovaTV 201 (NCT02001623), a phase 1-2, open-label, dose-escalation and dose-expansion clinical trial, evaluated the efficacy of TV in relapsed, advanced, or metastatic cancer of different types, including PROC [106]. TV demonstrated a manageable safety profile and promising preliminary antitumor activity in heavily pretreated patients across various cancer types. Therefore, future investigation into the optimal use of TV in solid tumors is justified [106].

Further, InnovaTV 208 trial (NCT03657043), a multi-center, open-label, phase II study assessed the safety and antitumor efficacy of TV in patients with platinum-resistant disease, primary peritoneal cancer, or fallopian tube cancer. The study has concluded, but its results have not yet been published. Therefore, future clinical trials are needed to fully establish TV as a therapeutic ADC for the treatment of ovarian cancer, including its long-term efficacy and safety profile [107].

## Challenges, recent advances, and future directions in the therapeutic use of ADC

Although ADCs hold promise for superior therapeutic benefits, several challenges must be addressed to ensure their optimal use in cancer treatment. One primary concern is toxicity management, as ADCs can cause off-target effects, especially when the drug payload is highly potent [108, 109]. The stability of the linker between the antibody and cytotoxic drug is also a crucial consideration, as an unstable linker may result in premature drug release, leading to increased toxicity. For example, in one of the clinical trials where MIRV (NCT01609556) was used, ocular treatment-related adverse events, such as reversible blurred vision and/or keratopathy, were observed [61, 62]. Similarly, in a phase I clinical study (NCT01911598) with LIFA tested in NaPi2b-expressing PROC patients, most common adverse effects included fatigue, nausea, decreased appetite, vomiting, and peripheral sensory neuropathy [95]. In another trial evaluating LIFA in combination with carboplatin (with or without bevacizumab) for recurrent platinum-sensitive ovarian cancer (PSOC), 83% of patients experienced grade ≥ 3 and 22% experienced serious TRAEs. Further, pulmonary toxicity was observed in 34% of cases, leading to one patient discontinuing the treatment due to grade 2 pneumonitis [97].

Similarly, in a phase I/II IMMU-132-01 basket trial (NCT01631552), where SG was evaluated for safety and efficacy in various solid tumors, including advanced OC, the trial was permanently discontinued due to adverse effects. The most common TRAEs were nausea (62.6%), diarrhea (56.2%), fatigue (48.3%), alopecia (40.4%), and neutropenia (57.8%). Serious adverse effects included febrile neutropenia (4.0%) and diarrhea (2.8%), with one treatment-related death from aspiration pneumonia [79]. Thus, the severity of side effects observed with these ADCs at the administered dose is prompting further investigation to optimize dosing to improve safety and efficacy.

Tumor heterogeneity also presents a challenge, as varying antigen expression among cancer cells limits the extent of targeting and therapeutic efficacy of ADC [110]. For example, heterogeneous expression of FOLR1, MSLN, and HER2, among others, has been observed in OC tumors, thus impacting the overall efficacy of these antigen-targeted therapies in the clinic [111–113]. Moreover, the lack of generalizability of the trial population limits information on the treatment’s efficacy and safety in the broader population. For example, in a clinical trial (NCT04209855), where MIRV was used for the treatment of platinum-resistant, high-grade serous ovarian cancer, eligibility criteria affected the generalizability of the trial population by restricting enrollment to participants who had previously received one to three lines of systemic therapy and excluding patients with primary refractory disease [68]. Generalizability could also be limited by a lack of racial and ethnic diversity. Such limitations underscore the importance of designing more inclusive clinical trials to ensure that findings are applicable to a wider range of patients.

A major challenge to the effectiveness of ADCs is the development of resistance through multiple mechanisms. These include alterations in target antigen presentation, such as reduced expression, structural mutations, or alternative splicing that hinders ADC binding. Resistance can also arise from impaired internalization of ADCs due to stromal or extracellular matrix barriers, as well as defects in endosomal trafficking or lysosomal function, limiting payload release. Additionally, increased drug efflux, metabolic modification of the payload, and instability in the tumor microenvironment can further reduce its efficacy. Many tumors upregulate DNA repair pathways or alter apoptotic signaling, enabling them to survive despite ADC-induced damage [114]. Addressing these challenges is important to improve efficacy and durability of ADC response in treatment of ovarian cancer.

Additionally, the lack of information regarding genomic markers that could regulate the response to ADCs further complicates treatment outcomes. For instance, the trial (NCT02751918) with Anetumab Ravtansine, an MSLN-targeting ADC used for epithelial platinum-resistant recurrent ovarian cancer and fallopian tube cancer, faced due to insufficient data on genomic markers that could influence response or resistance to the treatment [83]. This highlights the need for more detailed biomarker analyzes. Furthermore, small trial sizes and limited patient data contribute to the lack of comprehensive understanding of ADC efficacy. For example, a trial involving Tamrintamab Pamozirine (SC-003), an ADC targeting DPEP3, used in clinical trial NCT02539719 for the treatment of epithelial ovarian cancer (EOC), was unable to draw conclusions about its combination with budigalimab due to the limited patient sample size and data [103]. Similarly, the trial investigating Tisotumab Vedotin, an ADC targeting tissue factor for ovarian cancer, also had a small number of patients, limiting its findings [106]. Another example is Trastuzumab deruxtecan (T-DXd), an ADC targeting HER2, which is being studied in ovarian cancer treatment under trial NCT04482309, also has limited data available specifically for ovarian cancer [92]. Moreover, the complex and costly production processes of ADCs limit their availability and contribute to the small sample sizes and restricted data in clinical studies [115]. DCs may also elicit immune responses that affect both their safety and therapeutic potential, further constraining their clinical use [116, 117].

Although ADCs face several challenges in effective clinical use, recent advances in ovarian cancer treatment suggest that combining ADCs with other therapeutic agents could enhance their safety and efficacy. Strategies include pairing ADCs with immune checkpoint inhibitors, traditional chemotherapies, or targeted therapies like PARP inhibitors to improve response and combat resistance [118]. Preclinical and early-phase clinical trials have shown that such combinations, as previously described for various ADCs, could lead to synergistic effects, improving overall survival and minimizing toxicity compared to monotherapy [119]. Moreover, utilizing ADCs alongside targeted molecular therapies or agents that modulate tumor microenvironments could enhance the delivery and efficacy of ADCs [118].

Furthermore, novel ADCs with alternative warheads, including immune-modulating and DNA-targeting agents, showing promise in addressing resistance and off-target toxicity, could also be evaluated in clinical trials to improve efficacy [120, 121]. Additionally, improving ADC safety through advances in linker technology to enhance the stability and controlled release of warheads, and ensure that they remain inert in circulation but become active within tumor cells, will also facilitate the successful clinical use of ADCs [122]. Nevertheless, ongoing preclinical and clinical studies are essential to optimize these approaches and maximize ADC potential.

## Conclusion

ADCs offer a promising alternative to conventional therapies for OC, especially in platinum-resistant OCs. While clinical progress is evident, issues like antigen heterogeneity and drug resistance remain significant hurdles. Continued research into ADC optimization and combination therapies will be the key to improving patient outcomes and overcoming these challenges.

## Outstanding questions

- What strategies can be employed to overcome antigen heterogeneity in ADC therapies to improve their efficacy in treating platinum-resistant ovarian cancer?
- How can the development of next-generation ADCs address the challenges of off-target toxicity and minimize adverse effects in patients?
- What specific mechanisms drive ADC resistance in ovarian cancer, and how can these be targeted to enhance treatment outcomes?
- What are the key differences in clinical outcomes between ADCs targeting FOLR1, TROP-2, MSLN, NaPi2b, and HER2 in ovarian cancer, and how can these be leveraged to optimize therapy selection for individual patients?
- What are the most critical factors required in transitioning ADC-based therapies from early-phase trials to broader clinical use?
- How can combination therapies, such as ADCs with immunotherapies or targeted agents, be used to overcome the challenges of resistance in platinum-resistant ovarian cancer?
- What are the potential long-term impacts of ADC therapy on patient survival and quality of life in ovarian cancer, particularly in the context of platinum resistance and tumor heterogeneity?
